# Reducing central line associated bacteraemia in intensive care units using low cost strategies

**DOI:** 10.1186/1753-6561-5-S6-P58

**Published:** 2011-06-29

**Authors:** M-L Mclaws, A Burrell

**Affiliations:** 1 Public Health and Community Medicine, University of New South Wales, Sydney, Australia; 2 Patient Safety, Clinical Excellence Commission, Sydney, Australia

## Introduction / objectives

Aseptic insertion technique of central lines has reduced Central line associated bacteremia (CLAB) using bundling of low cost patient and physician preparation strategies. Bundle compliance rates and survival analysis were used to illustrate improvements and gains in risk-free line-days.

## Methods

36 adult ICUs in New South Wales, Australia implemented *aseptic insertion bundle*s over 18-months. Patient bundle included 2% alcoholic chlorhexidine preparation of insertion site, full sterile sheet drape, line position checked with x-ray and/or transducer. Physician bundle included hand hygiene, hat, mask, eyewear, sterile gloves/gown, maintain sterile technique. The first 12-month roll-out period was used to train all ICU physicians to implement the bundles and collect data. 10,575 lines inserted were followed until discharged from ICU. CLAB rates and bundle compliance rates were calculated quarterly and survival analysis established the CLAB-free line-day period.

## Results

The CLAB rate was significantly reduced from 3.0/1000 line-days in the first 12-months to 1.2/1000 line-days in the last 6-months (P=0.0006). Compliance with both physician and patient bundles reduced CLAB (RR 0.5, 95%CI 0.4-0.8, P=0.004) compared with non compliance while non compliance with the physician bundle increased the risk of CLAB (RR 1.62, 95%CI 1.1-2.4, P=0.0178). The CLAB rate was 3.8/1000 (95%CI 2.5-5.5) line-days in the first 12-months and was significantly reduced by the last 6 months (1.6/1000 line-days, 95%CI 1.0-2.4). CLAB commenced at day-7 (1.8/1000 line days) in the first 12-months but did not occur until day-9 (0.9/1000 line-days) by the last 6 months.

## Conclusion

CLAB is essentially preventable with a highly effective low cost aseptic insertion technique that enables patients to remain risk-free for the first 9 line-days.

## Disclosure of interest

None declared.

